# A Framework for Online Experimenter-Moderated Looking-Time Studies Assessing Infants’ Linguistic Knowledge

**DOI:** 10.3389/fpsyg.2021.703839

**Published:** 2021-09-24

**Authors:** Desia Bacon, Haley Weaver, Jenny Saffran

**Affiliations:** Infant Learning Lab, Department of Psychology, Waisman Center, University of Wisconsin-Madison, Madison, WI, United States

**Keywords:** infancy, online, eye-gaze, methodology, diversity, recruitment

## Abstract

Online data collection methods pose unique challenges and opportunities for infant researchers. Looking-time measures require relative timing precision to link eye-gaze behavior to stimulus presentation, particularly for tasks that require visual stimuli to be temporally linked to auditory stimuli, which may be disrupted when studies are delivered online. Concurrently, by widening potential geographic recruitment areas, online data collection may also provide an opportunity to diversify participant samples that are not possible given in-lab data collection. To date, there is limited information about these potential challenges and opportunities. In Study 1, twenty-one 23- to 26-month-olds participated in an experimenter-moderated looking-time paradigm that was administered *via* the video conferencing platform Zoom, attempting to recreate in-lab data collection using a looking-while-listening paradigm. Data collected virtually approximated results from in-lab samples of familiar word recognition, after minimal corrections to account for timing variability. We also found that the procedures were robust to a wide range of internet speeds, increasing the range of potential participants. However, despite the use of an online task, the participants in Study 1 were demographically unrepresentative, as typically observed with in-person studies in our geographic area. The potentially wider reach of online data collection methods presents an opportunity to recruit larger, more representative samples than those traditionally found in lab-based infant research, which is crucial for conducting generalizable human-subjects research. In Study 2, microtargeted Facebook advertisements for online studies were directed at two geographic locations that are comparable in population size but vary widely in demographic and socioeconomic factors. We successfully elicited sign-up responses from caregivers in neighborhoods that are far more diverse than the local University community in which we conduct our in-person studies. The current studies provide a framework for infancy researchers to conduct remote eye-gaze studies by identifying best practices for recruitment, design, and analysis. Moderated online data collection can provide considerable benefits to the diversification of infant research, with minimal impact on the timing precision and usability of the resultant data.

## Introduction

Developmental researchers face a multitude of barriers to completing research, particularly in determining methodologies appropriate for measuring various cognitive phenomena and participant recruitment. In particular, infants cannot provide verbal responses to interrogate underlying cognitive processes and thus researchers must rely on implicit behaviors such as eye-gaze. Moreover, infant samples are difficult to recruit and subject to high attrition rates (Enders, 2013; Klein-Radukic and Zmyj, 2015; Nicholson et al., 2015), resulting in local convenience sampling and limited generalizability. The coronavirus pandemic further complicated developmental work by limiting feasible in-person methodologies. The availability of well-defined virtual data collection methods for use with infants and young children is limited compared with adult methods, creating delays for research programs that rely on methods like eye-gaze. For many common infant cognition tasks, eye-gaze behavior is coded relative to audio stimuli presentation, which necessitates that the timing of stimulus presentation and data uptake must be quite accurate. Families may vary in the speed and reliability of their home internet connection, which can have downstream impacts on the timing of stimulus presentation and the frame rate of video recordings. In fact, prior research using videoconferencing describes internet connectivity, stability, and video quality as some of the disadvantages to collecting data virtually (Archibald et al., 2019). This may be particularly challenging for studies focused on language learning due to the need to integrate audio-visual stimuli. For example, to assess word recognition using eye-gaze behavior, researchers analyze visual attention to a particular image after hearing the onset of a word. Thus, inferences in these paradigms crucially depend on the temporal alignment of looking behavior to the onset of an auditory stimulus.

Existing online methods for developmental research are primarily geared toward either unmoderated data collection or older children. LookIt, an infant and child research platform based at MIT, allows researchers to upload unmoderated experiments to the platform to be completed by participants and caregivers (Scott and Schulz, 2017). TheChildLab is an experimenter-mediated video chat platform for study administration used with slightly older populations (aged 5+; Sheskin and Keil, 2018). Using TheChildLab, Sheskin and Keil (2018) were able to replicate in-lab effects for this age group. Both LookIt and TheChildLab demonstrate the feasibility of doing online research with developmental populations, though only a limited set of tasks have been verified for use *via* online platforms.

In addition to these platforms, developmental researchers have been using Zoom for experimenter-mediated studies. For example, Smith-Flores et al. (2021) used Zoom to replicate several in-lab findings using violation of expectation paradigms with 15-month-old. Importantly, Smith-Flores et al. (2021) reported global measures of looking-time (i.e., average proportion of looks), but did not examine moment-by-moment changes in visual attention in response to a stimulus, as is common with lab-based experiments focused on early language development. Although Sheskin and Keil (2018) and Smith-Flores et al. (2021) suggest that experimenter-moderated data collection is promising, it is unclear how variability between participants’ home set-ups impacts the subsequent data quality. Lack of internet access or poor internet connectivity could render participants’ video data unusable due to inconsistent stimulus presentation. However, limiting participation to only high-speed internet users could create a significant barrier to families’ ability to participate in online studies further perpetuating the issue of infant samples drawn predominantly from highly educated and wealthy families. Despite the unknown variability in home-set ups and internet access, the use of online data collection methods could provide an opportunity to ameliorate another persistent problem in developmental research: the lack of diversity of infant participants in lab-based studies.

Psychological research with human participants has historically relied on White, upper-, to upper-middle-class convenience samples. The resulting findings are representative of the participant group, but not necessarily of the wider, more diverse population the results are often applied to. Several research bodies have long recommended diversification of both researchers and participants in psychological research, placing the onus on the researchers to recruit members of underrepresented groups (National Institutes of Health, 1994; American Psychological Association, 2003; American Psychological Association, APA Task Force on Race and Ethnicity Guidelines in Psychology, 2019). Despite this push from respected institutions like the NIH and APA, psychological research has continued to primarily consist of Western, Educated, Industrialized, Rich, and Democratic (WEIRD; Henrich et al., 2010) convenience samples, tested at or in the immediate areas around universities. There has been a push for researchers to report their diversity (or lack thereof) in their research proposals and publications. Many proposals and publications report that their sample is representative of the local population; however, simply matching local census proportions does not make results generalizable. The geographic locations of universities and their surrounding population demographics place limitations on the population that can access in-person studies. The internet, and its increasingly pervasive presence in homes around the world, presents the opportunity to reach a more diverse participant sample.

Online recruitment and research with adult participants support the assertion that online data collection can lead to a diversification of participants. In adult studies, Amazon Mechanical Turk (MTurk) samples provide the most ethnic and socioeconomic (SES) diversity out of all of the adult study platforms (Casler et al., 2013), though it is not the only platform that works to recruit diverse samples (Casler et al., 2013; Buhrmester et al., 2016). Importantly, data quality was similar across participants regardless of whether they were recruited and participated *via* MTurk, social media, or face-to-face behavioral testing (Casler et al., 2013). These adult findings provide hints that variability in home data collection environments can have beneficial impacts on diversity without significant differences in experimental results. By extending recruitment efforts and increasing the diversity of participant samples through online methods, results are more representative across race and SES.

Online recruitment methods, although able to reach a wider audience than typically is reached in the community surrounding universities, are not without their own impediments. Facebook, one of the most widely used digital recruitment platforms, is much more popular with White users, while Instagram is more popular with Latinx and Black users (Krogstad and Pew Research Center, 2015). The 2012 Facebook acquisition of Instagram, and the integration of the ad features on both platforms, allows ads that originate on one platform to appear in feeds of users on the other platform. As of August 2020, Facebook no longer allows demographic information pertaining to race to be used in targeted ads. Displaying the same ad across platforms may ameliorate disproportionate ad display to specific racial groups. Researchers working with adults have successfully increased the racial diversity of their samples without race-based microtargeting by targeting zip codes with larger non-White populations while keeping other targeted features constant (i.e., targeting people with particular sets of interests; Pechmann et al., 2020). While these approaches work well for adult samples (Casler et al., 2013; Pechmann et al., 2020), it is unclear whether a similar digital approach to recruitment and study administration is plausible for infant studies.

There is some evidence that online methods of recruitment targeting parents could be effective at recruiting more diverse infant and child participants for developmental research. Recruitment of parents *via* MTurk is fast, cheap, and results in more diversity than relying on Listservs (Buhrmester et al., 2016; Dworkin et al., 2016). Facebook ads targeting parents of specific races and ethnicities also yield more diversity than relying on Listservs or posting flyers around communities (Dworkin et al., 2016; Jang and Vorderstrasse, 2019). The LookIt platform provides a more racially diverse and representative United States participant sample than in-lab studies (Scott and Schulz, 2017). Although online data-sharing platforms, like the Databrary Project^1^ and the Child Language Data Exchange System (CHILDES) allow researchers to access data from studies conducted globally (MacWhinney, 2000; Adolph et al., 2017), developmental researchers have called for greater efforts to conduct studies with representative samples. In particular, several scholars have proposed the development of Collaboration for Reproducible and Distributed Large-Scale Experiments (CRADLE) where there can be a combining of data from multiple data collection sources, addressing the need for more diverse and inclusive samples (Sheskin et al., 2020).

The present studies aim to address gaps in our understanding of online data collection and recruitment methods by (1) evaluating whether looking time data collection and retention with infants is possible across a range of home-set up variables and (2) investigating whether online data collection can facilitate more representative research by recruiting more diverse potential participants.

To determine whether real-time eye-gaze behavior can be captured in a Zoom study protocol, Study 1 includes a standard looking-while-listening (LWL) procedure with static images (Fernald et al., 2008). In face-to-face lab tasks, this method typically uses an eye-tracker to collect data, though there is evidence that hand-coding the data from video using custom software is not only reliable, but actually yields more usable trials and larger effect sizes than remote eye-tracker data (Venker et al., 2020). Study 1 was designed to test the hypothesis that experimenter-moderated studies over Zoom yield high quality infant data using LWL. The session recordings used for our primary method of data collection are the result of participant screen-sharing, which leaves the data subject to several uncontrolled variables: the frame rate of Zoom, the internet upload speed of the participant’s computer, and the internet download speed of the experimenter’s computer. Thus, a central goal of Study 1 is to evaluate these factors to determine whether variability in home set-up hinders interpretation of looking time data collected online. To assess the quality of the data, we examine the time course, average speed (reaction time, RT), and accuracy of looks to the targets during the LWL task. We compare these measures of data quality to a sample of data from Peekbank (Zettersten et al., 2021), an open-source database of in-lab LWL studies. We predict that experimenter-moderated Zoom LWL will approximate in-person data collection in timing precision and word recognition accuracy.

**Figure 1:**
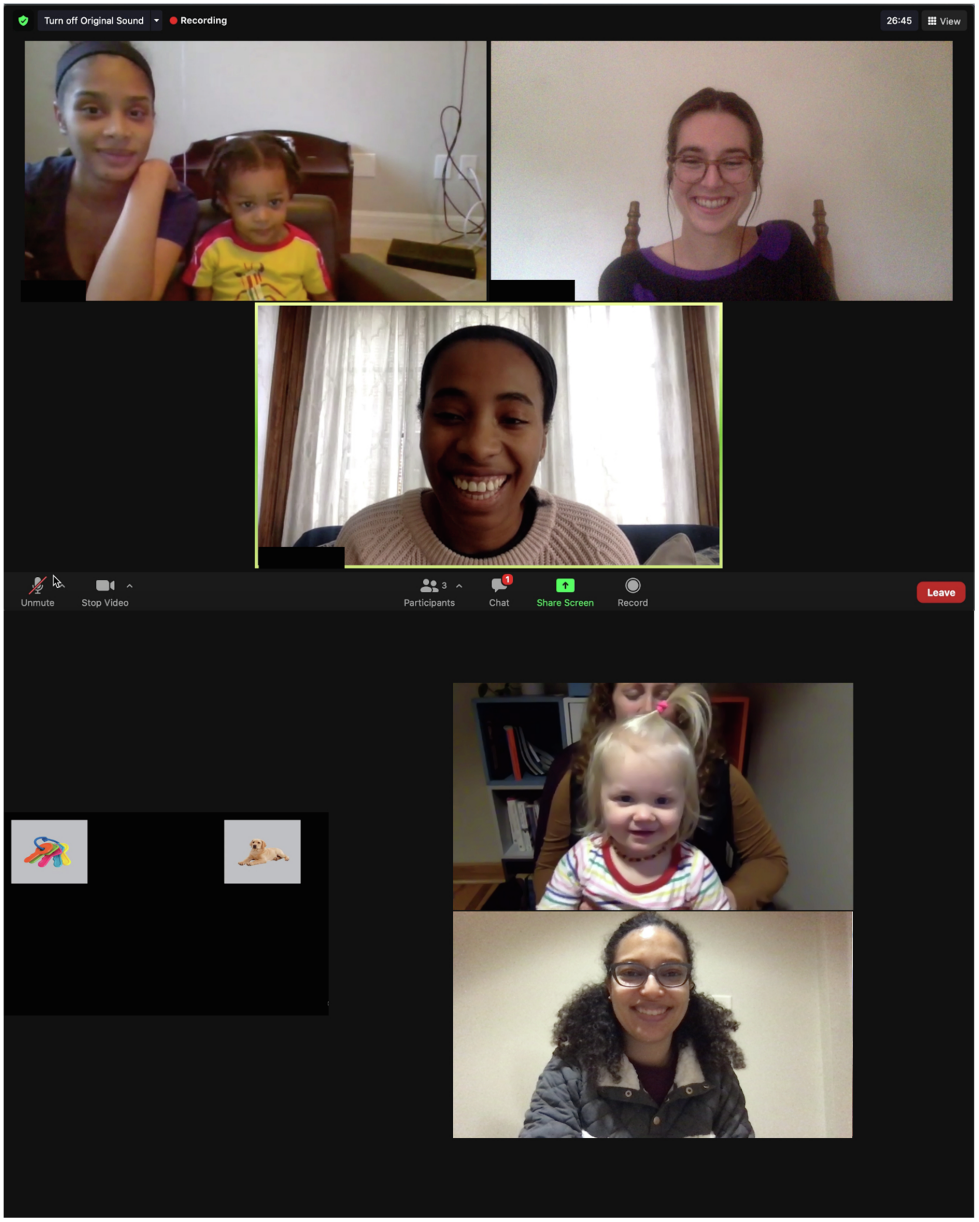
Facebook advertisement used for microtargeting.

While Study 1 provided insight into the validity of online eye-gaze data across a range of set ups, the study did not adequately address diversity initiatives assumed to be improved using online data collection. Indeed, the participants in Study 1 were no more diverse than those we typically see for in-lab studies in our community. Thus, Study 2 was designed to determine if online recruitment targeting more diverse geographic locations could increase the diversity of participant signups for future study participation. Specifically, we asked whether more diverse families than those in the surrounding local population would express interest in participating in online experiments as a result of a microtargeted social media advertisement. We selected two locations in the same US state and matched them to be comparable in population size. Site 1 was predominantly Black and lower SES, and Site 2 was predominantly White and higher SES. We created a single ad using photos of lab participants and experimenters during a mediated online study session; the photos depicted racial diversity in both the participants and the experimenters (see Figure 1). Using Facebook’s system for creating ads, we targeted the ad to the zip codes of the two sites, and then added interest-based targeting details that were race-neutral (e.g., parenting and childbirth). The ad linked to a lab sign-up page where the families of potential participants could enter information to be contacted for future studies. Based on prior research suggesting the efficacy of using diverse targeted advertisements for recruitment, we predicted that we would obtain more diverse participant sign-ups when an ad is targeted to people living in a more diverse area (Dworkin et al., 2016; Jang and Vorderstrasse, 2019; Pechmann et al., 2020).

## Study 1: Online Looking Time Study

### Methods

Infants saw a pair of familiar objects on each trial and heard the speaker ask for a target item. We predicted that infant word recognition and performance on the LWL task would be comparable to the results of lab studies that use familiar nouns as stimuli accessible on Peekbank (Zettersten et al., 2021), a database of LWL studies. Thus, we expected infants to show an increase in target looking following the onset of the noun. However, note that in the current paper, we will focus on assessing data quality by testing the hypothesis that data collected *via* Zoom will approximate an in-lab sample from Peekbank.

#### Participants

Twenty-one full-term, monolingual English-learning infants (nine females) with a mean age of 25.0months (23.0–26.0) were included in the analyses. Families were recruited from an existing research database tied to the local community (*n*=19 identified as non-Hispanic White; *n*=1 identified as multiracial; and *n*=1 identified as Hispanic White). Caregivers reported that their children had no history of developmental concerns, heard fewer than 10h per week of another language, and were currently free of ear infections. Eight additional participants were excluded due to: technical error (*n*=4), experimenter error (*n*=2), or failure to complete the task (*n*=2). Caregivers provided written informed consent. All experimental protocols were approved by the local Institutional Review Board. Data were collected between 10/20 and 02/21 as part of a larger project investigating the relation between words and knowledge of object functions.^2^

#### Materials

A female native speaker of English recorded 12 sentences using infant directed speech. Each sentence included one of four carrier phrases (i.e., “Find the [target noun]!,” “Look at the [target noun]!”) followed by a target noun (*apple*, *ball*, *crayon*, and *toothbrush*). Still images of the target nouns were selected (Brodeur et al., 2014) and placed on a grey 360×360-pixel gray background using GIMP.^3^ Three unique images were chosen for each of the target nouns for a total of 12 still images. Each object occurred equally as often as a target and distractor.

#### Procedure

Participants were tested *via* the videoconferencing platform Zoom. Caregivers completed a home setup procedure guided by an experimenter to maximize their lighting, screen display, and child positioning. Caregivers used their own computer (laptop or desktop) to access a personalized study link and shared their screen with the experimenter for screen recording. Study participation was limited to those with access to a computer due to the inability to screenshare and the constraints on stimuli size when using tablets or smartphones. Caregivers closed their eyes during testing to minimize bias. Each Zoom session, including the caregiver’s shared screen displaying the experimental procedure, was recorded locally by the experimenter for offline eye-gaze coding, frame-by-frame (40ms), using an open-source program for eye-gaze coding (Peyecoder; Olson et al., 2020).

Infants’ real-time word comprehension was assessed using LWL (Fernald et al., 2008). On each trial, two pictures of familiar objects were displayed simultaneously in silence for 1,000ms. Stimuli were aligned horizontally at a fixed distance of 540 pixels, which was held constant across all participants regardless of screen size. Infants heard speech labeling one of the objects in a carrier phrase (767ms) ending in the target noun (708ms). Infants were allowed to view the images for 2,025ms after the offset of the target noun for a total trial length of 4,500ms. There were six test trials for each target noun for a total 24 test trials. Trials were presented in a pseudorandom order in blocks of six interspersed with attention getters.

##### Internet Speed Test

To evaluate the impact of internet variability, we simulated the participant and researcher experience with the online task under different internet speeds using the developer tools in Google Chrome [Version 90.0.4430.85 (Official Build; x86_64)]. We tested four different internet speeds (2G, 3G slow, 3G fast, and 5G no throttling) to verify that events occurring on the Zoom recording reflect the events that a participant experienced. The internet tests were used to ensure that the events captured within our Zoom recordings can reliably be time-locked to the participant’s eye-movements. They also provided independent verification that we could include data from a range of home set-ups and did not need to exclude participants on the basis of internet speed.

For each internet speed test, two researchers imitated the experimental procedure by deploying the task in Google Chrome while participating in a Zoom call. One of the researchers, serving as the “participant,” shared their screen. Two videos were recorded from each speed simulation. One video was recorded from the experimenter perspective using Zoom to imitate the data collected during an experimental session. The second video was a screen recording [QuickTime Version 10.5 (1015.2.1); recorded at 60fps] of the Google Chrome window running the experimental procedure from the participant perspective to capture a participant’s experience of the task at the current internet speed. A trained research assistant coded the trial onsets and offsets of the videos using Peyecoder (Olson et al., 2020).

##### Peekbank Data

In order to have a reasonable point of in-lab comparison for our experimenter-moderated online LWL task, we consulted Peekbank, a new open-source database of LWL studies (Zettersten et al., 2021). Using peekbankr, we searched for experiments testing infants in our target age range (23- to 26-month) whose primary language is English. We then filtered this sample for data testing familiar words (rather than nonce words). This yielded a sample of data from 126 participants across six studies (Yurovsky et al., 2013; Mahr et al., 2015; Frank et al., 2016; Yurovsky and Frank, 2017; Potter and Lew-Williams, in prep; Yurovsky et al., under review). One study was excluded for using a tablet-based LWL paradigm, which does not reflect our typical in-lab data collection paradigm using an eye-tracker. We also filtered the sample, limiting it to our specific target words (apple, ball, crayon, and toothbrush) of which only apple and ball were included in the dataset. Across three experiments (Mahr et al., 2015; Potter and Lew-Williams, in prep; Yurovsky et al., 2013), data from 70 participants for these two target words were obtained.

#### Coding

Trained research assistants coded eye movements frame-by-frame at a frame rate of 25fps using Peyecoder (Olson et al., 2020). Coders indicated whether infants were looking left, right, or off (i.e., in a gaze shift between images or looking off screen). Twenty-five percent of the videos were randomly selected and independently recoded. We evaluated reliability on three measures: (1) the percentage of gaze shifts that occur within a one-frame threshold (i.e., do coders agree on the timing of coded events?; shift agreement; 93.48%); (2) the percentage of event frames that have the same response between coders (i.e., do coders agree whether a frame is coded as left, right, or off?; frame agreement; 95.52%); and (3) the percentage of trials that have the same number of coded events between coders, impacting how many trials were used to calculate shift agreement (comparable trials; 85.68%).

### Results

#### Internet Speed Tests

For each internet speed (2G, 3G slow, 3G fast, and 5G no throttling), we calculated the total number of comparable trials and the frame agreement between the two videos to assess whether the number of trials captured by the Zoom recording differed from the participant’s experience of the study. If there is an internet lag, the number of trials seen in the participant view could differ from the experimenter view. Table 1 provides the frame agreement, number of trials, and trial response agreement between the experimenter and participant perspective videos. Regardless of internet speed, the experimenter and the participant videos aligned. When independently coded, the participant and experimenter videos at each speed level have the same number of frames, number of trials, and the same trial responses. Most importantly, the lack of frame disagreement suggests that internet speed is not a significant barrier for online participation in tasks using audio, images, and videos. Although slower internet speeds influenced the presentation of the experiment (Table 1), the recorded Zoom data capture this inconsistency, which allow for unpresented trials to be skipped during data analyses. The present results suggest that high-speed internet is not a prerequisite for usable data quality in online studies.

**Table 1:** Internet speed simulation.

	2G	3G slow	3G fast	5G no throttling
Frame agreement	100.0%	100%	100%	100%
Number of trials Participant: Experimenter	35:35^*^	36:36	36:36	36:36
Trial response agreement	100.0%	100%	100%	100%

The asterisk (*) denotes a different number of trials than the experiment total, illustrating trial loss.

#### Time Course of Looking Behavior

The aim of the looking time results is to introduce a new procedure for correcting eye-gaze data given variable frame rates, and to provide evidence that eye-gaze behavior timing information recorded online is interpretable and comparable to in-lab research gathered from Peekbank. We report data visualizations in the form of time course plots to visually assess whether the data approximate what would be expected from data collected in the lab. In particular, we plot the proportion of looks to the target as a function of time with confidence bands reflecting SE of the point estimate. No inferential statistics were conducted.

Infant looking behavior was coded frame-by-frame resulting in eye-gaze data every 40ms over the course of a trial. We computed the proportion of looks to the target visual stimulus [accuracy; looks to target/(looks to target+looks to distractor)] at each 40ms time bin averaging across trials and participants. We were interested in looks beginning at 300ms after the onset of the target word and ending 1,800ms after target word onset (Fernald et al., 2008). The target window was selected to reflect similar window of analyses used in prior LWL studies with toddlers (Swingley and Aslin, 2000; Swingley and Aslin, 2002; Fernald et al., 2008; Bergelson and Swingley, 2012; Zettersten et al., 2021). We excluded trials that did not include looks to either the target or distractor image for at least 50% of the frames. Across all participants, only 59 trials out of the total 504 trials were excluded using this criterion (i.e., 88% of trials were usable). Individual infants contributed an average of 21 useable trials (range: 15–24) out of a maximum 24 trials in the study, with no infant contributing fewer than 50% of all trials.

To evaluate the reliability of timing data derived from Zoom recordings, we examined whether the number of frames per trial replicated the expected total number of frames given the length of a trial. A coded LWL trial was 3,900ms and therefore should include 98 frames of looking data (3,900ms/40ms per frame). For each infant, we calculated the number of frames recorded for each LWL trial. On average, there were 92 frames per trial (range=20–136), which is six frames less than expected given the trial length. Therefore, the average time elapsed per frames is longer (43.86ms) than the assumed 40ms frame rate of Zoom recordings. There is an inverse relationship between ms per frame and the number of frames in a trial, such that longer frame lengths indicate a fewer total number of frames on a given trial. This timing discrepancy has implications for data coding. In particular, if the onset of a target noun is expected to occur at frame 29 (1,167ms onset time/40ms per frame), then it is actually occurring at frame 27 on average (1,167ms onset time/43.86ms per frame) due to the longer average length of a frame. Furthermore, the difference in the number of ms per frame can vary from trial to trial for a given participant, with the length of a frame ranging from 28.69 to 195ms. Therefore, for some trials, the onset of the target word occurs later in the trial, at frame 40, while for others it could occur as early as frame 6.

Given the discrepancy between expected and actual frame rates, we plotted the time course of target looks using two different measures of time: (A) uncorrected time using the Peyecoder frame rate (40ms) and (B) corrected time using each infant’s average frame rate. For each infant, we computed the frame rate for each trial by calculating the average number of ms that elapsed per frame (i.e., a frame rate of 25fps indicates that 40ms elapses per frame). We calculated the length of a frame (in ms) by dividing the total length of a trial (3,900ms) by the total number of frames within each trial. Each child’s timing data were adjusted frame-by-frame using their by-trial frame rate. For example, an event occurring at frame 29 in the assumed frame rate of 40ms per frame (based on the Peyecoder output) would be adjusted to occur at frame 31 for an infant who had an actual frame rate of 37ms per frame. To normalize the data across participants, we calculated the mean frame rate by averaging across all trials contributed by all participants. The adjusted timing data were binned into 43.86ms increments (22.80fps) to have comparable time bins across infants. Thus, a looking event that occurred at an assumed 40ms was adjusted to occur at an actual 43.86ms. Normalizing the timing data in this manner results in 90 frames that increment in 43.86ms time windows from 0 to 3,900ms. This process ensured that trials with different frame rates could still be averaged together to yield group level looking accuracy across infants and across trials.

**Figure 2:**
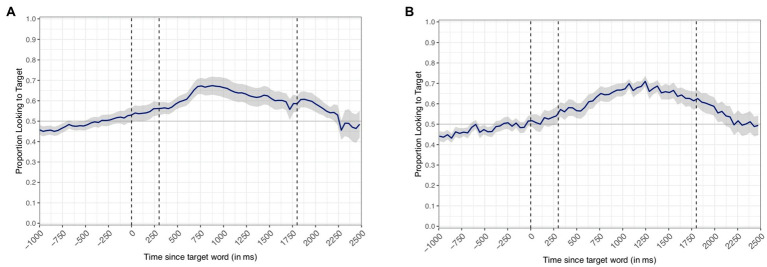
Proportion of looks to a target image as a function of time. Dashed lines represent the onset of the target word (0ms) and demarcate the primary window of interest from 300 to 1,800ms. Graph **(A)** plots uncorrected time using the expected frame rate of 40ms per frame (25fps). Graph **(B)** plots time that has been corrected to reflect each individual infant’s mean-adjusted frame rate and normalized across participants to a frame rate of 43.84ms per frame. Confidence bands represent SEM.

The results of the adjusted time course of looks, collapsed across participants, can be seen in Figure 2. Notably, infants increased the proportion of looks to the target item during the critical window from 300 to 1,800ms in both plots, suggesting that infants recognized the target words. However, the corrected time course plot shown in Figure 2B demonstrates that when adjusting individual participants’ data timing into the same time bins (using averaged frame rate) and then collapsing across participants, the looking behavior shows a looking pattern more similar to in lab eye-gaze assessment. Specifically, the accuracy in looks to the target in Figure 2B begins to diverge from chance closer to 300ms (i.e., the approximate time it takes to execute a planned eye movement based on phonological information; for discussion of latencies to shift in LWL see Swingley and Aslin, 2000; Fernald et al., 2008; Zettersten et al., 2021) after the onset of the target noun as compared to Figure 2A, in which accuracy differs from chance beginning at approximately 150ms. Further, Figure 2A reflects what would be expected by plotting timing data that results from a slower frame rate because the target word onset actually occurs in an early frame. The results of the time course plots suggest that online data collection can replicate previous findings for familiar word recognition (Fernald et al., 1998; Bergelson and Swingley, 2012; Bergelson and Aslin, 2017) despite some limitations due to variable frame rates.

**Figure 3:**
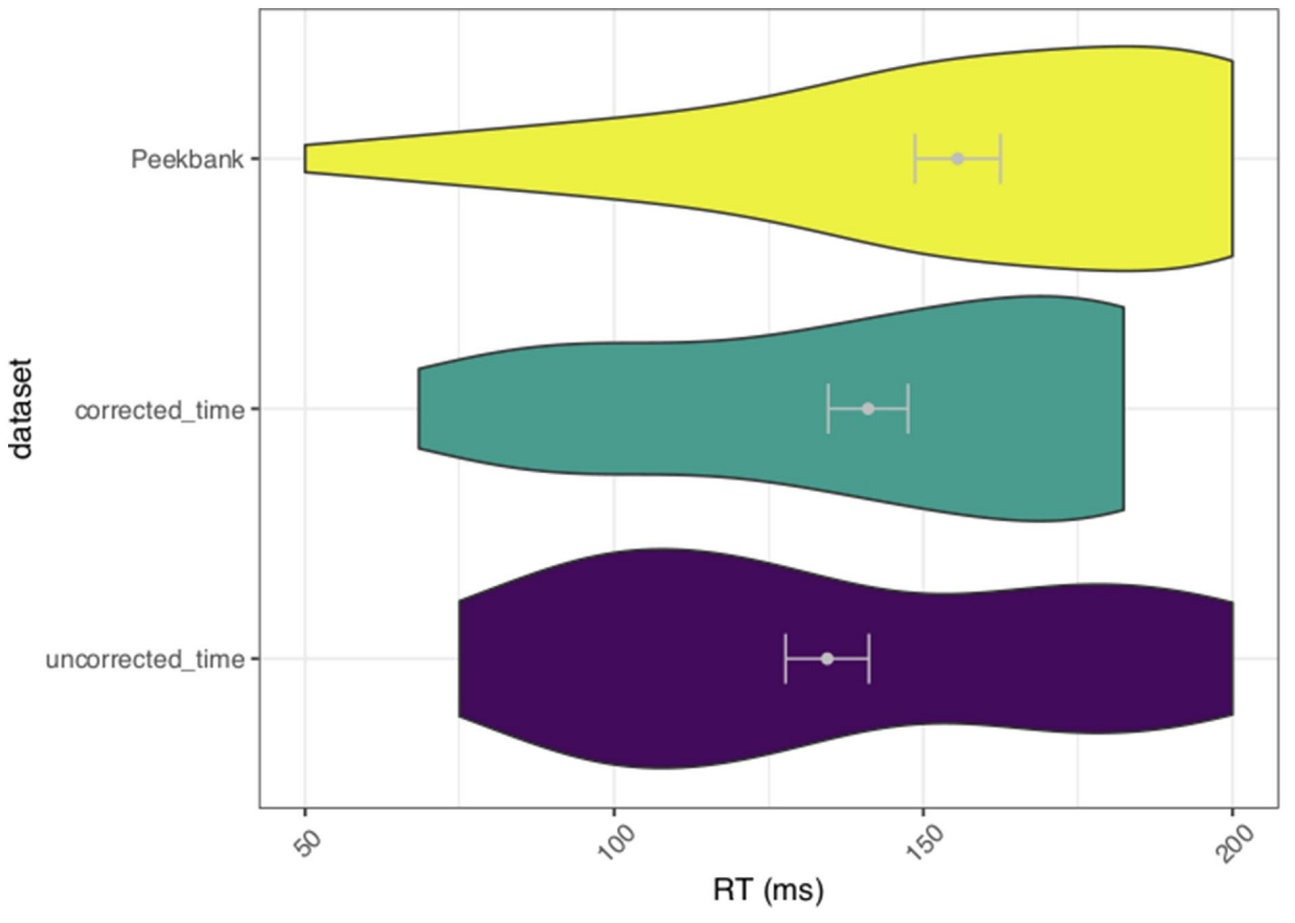
Average latency to shift to the target from distractor during a window from −300 to 200 between Peekbank data and the Study 1 corrected and uncorrected timing data. Error bars represent SEM.

The time course of target looks in the uncorrected data suggests that infants are shifting earlier than what would be expected in response to the auditory stimulus. We thus wanted to determine whether our process for timing corrections would more closely approximate the shift latencies in a sample of data from similar tasks collected in-person. Thus, we assessed whether there were significantly later shifts to the target in LWL data collected in person compared to the current sample of data collected *via* Zoom. Based on the time course of looks seen in Figure 2, we defined a window of analysis from −300 to 200ms. This analysis window reflects a period of time when the average curve in the uncorrected timing data begins to deviate from chance to a point in time when the confidence bands do not include chance responding (Figure 2). Importantly, this time period occurs earlier than we would normally expect to see eye gaze behavior in response to the spoken words with most studies approximating looking behavior to begin around 300ms (e.g., Fernald et al., 1998; Swingley and Aslin, 2002; Fernald et al., 2008; Garrison et al., 2020). We expected that in-person LWL data, as represented by a Peekbank sample, would have significantly later shifts to the target than the uncorrected Zoom LWL data. However, if our adjusted time course is a more veridical representation of the task, we would expect that the in-person LWL data would not differ from the corrected Zoom LWL data. To test this hypothesis, we identified all trials in which an infant was fixating the distractor at the onset of the analysis window. We then calculated the latency to shift to the target image. We fit a linear mixed effects model (LMEM) predicting shift latency from the different datasets (i.e., uncorrected Zoom data, corrected Zoom data, and Peekbank data) including a by-subject random intercept and a by-item random intercept. We coded Peekbank as the reference group to compare whether the data collected *via* Zoom differed significantly from data collected using LWL in-lab. The average latency to shift was significantly later (*M*=155.556) in the Peekbank dataset compared to the uncorrected LWL data collected *via* Zoom [*M*=134.460; *b*=−34.997; *t*(1, 39.697)=−2.224; *p*=0.032; 95% CI (−65.844, −4.151)]. There was no significant difference in timing between the Peekbank dataset and the corrected LWL Zoom data [*M*=141.074; *b*=−16.069; *t*(1, 52.328)=−0.939; *p*=0.352; 95% CI (−49.601, 17.464); Figure 3]. This analysis supports our contention that the timing correction serves an important data preprocessing step in adjusting the timing of the trial so that it more accurately reflects the actual presentation of stimuli.

#### Reaction Time Results

We were interested in evaluating how the timing of shifts in response to an auditory stimulus in the present Zoom study compares to an in-person LWL designs. To examine this question, we compared the average reaction time (RT) for the uncorrected and corrected timing data to samples of LWL data from Peekbank (Zettersten et al., 2021) that test a subset of the target words (i.e., apple and ball) in our target age range (23- to 26-month). RTs were defined as the average time it takes an infant to shift from the distractor image to the target image on a given trial from the onset of the target word (0ms) to 1,800ms post-word onset (Fernald et al., 2008). We would expect that the RTs calculated using the corrected timing data should be more similar to samples drawn from Peekbank than the uncorrected timing data.

Reaction time was calculated for all trials in which an infant was initially fixating the distractor at the onset of the target time window. For each trial, we calculated an infant’s latency of the first shift to the target image from the distractor image (Fernald et al., 2008). We then filtered out RTs that were later the predetermined window length. This definition of RT does not include time to fixate the target, but rather demonstrates the time it takes to process the auditory stimulus and make a behavioral response.

We fit a LMEM to compare RTs within the target window (0–1,800 ms) to assess average shift latency in response to the target words. We regressed RTs on a variable for the different datasets including a by-subject random intercept and slope for dataset and a by-item random intercept and slope for dataset. Dataset was contrast coded using Peekbank as the reference group to assess whether the average RTs from the corrected and uncorrected Zoom data differ significantly from RTs typically seen in-lab. RTs from the Peekbank dataset were significantly longer (*M*=909.420) than both the corrected [*M*=417.747; *b*=−550.366; *t*(1, 6.357)=−7.525, *p*<0.05; 95% CI (−693.723, −407.010)] and uncorrected online data [*M*=399.722; *b*=−571.914; *t*(1, 5.276)=8.067; *p*<0.05; 95% CI (−710.866, −432.963)]. It is possible that the methodological differences between in-lab and online studies (i.e., screen size, distance from the monitor, and number of test trials per word) could account for faster RTs in the online experiment. We return to this possibility in the Study 1 Discussion.

#### Accuracy Results

We were interested in whether the data collected *via* Zoom would approximate word recognition accuracy for familiar words that is expected from in-lab LWL designs. Thus, we compared the average proportion of looks to the target image (accuracy) in the corrected online timing data to the sample of LWL data from Peekbank. For each dataset, we computed an infant’s by-trial average accuracy during the window from 300 to 1,800ms (Fernald et al., 2008). If the mode of data collection has minimal impact on the resultant data quality, we would expect minimal differences in accuracy across these study types.

We fit a LMEM regressing accuracy on dataset type including a by-subject random intercept and a by-item random intercept. We also included an offset at 0.5 to evaluate whether average accuracy differed significantly from chance responding. For this analysis, the corrected online dataset served as the comparison group (coded as 0) to determine whether the datasets derived from in-lab studies (Mahr et al., 2015; Potter and Lew-Williams, in prep; Yurovsky et al., 2013; Yurovsky and Frank, 2017; Yurovsky et al., under review) differed significantly in average accuracy for familiar word recognition from the current study. We report Holm-Bonferroni corrected values of *p* to account for multiple comparisons (Holm, 1979).

**Figure 4:**
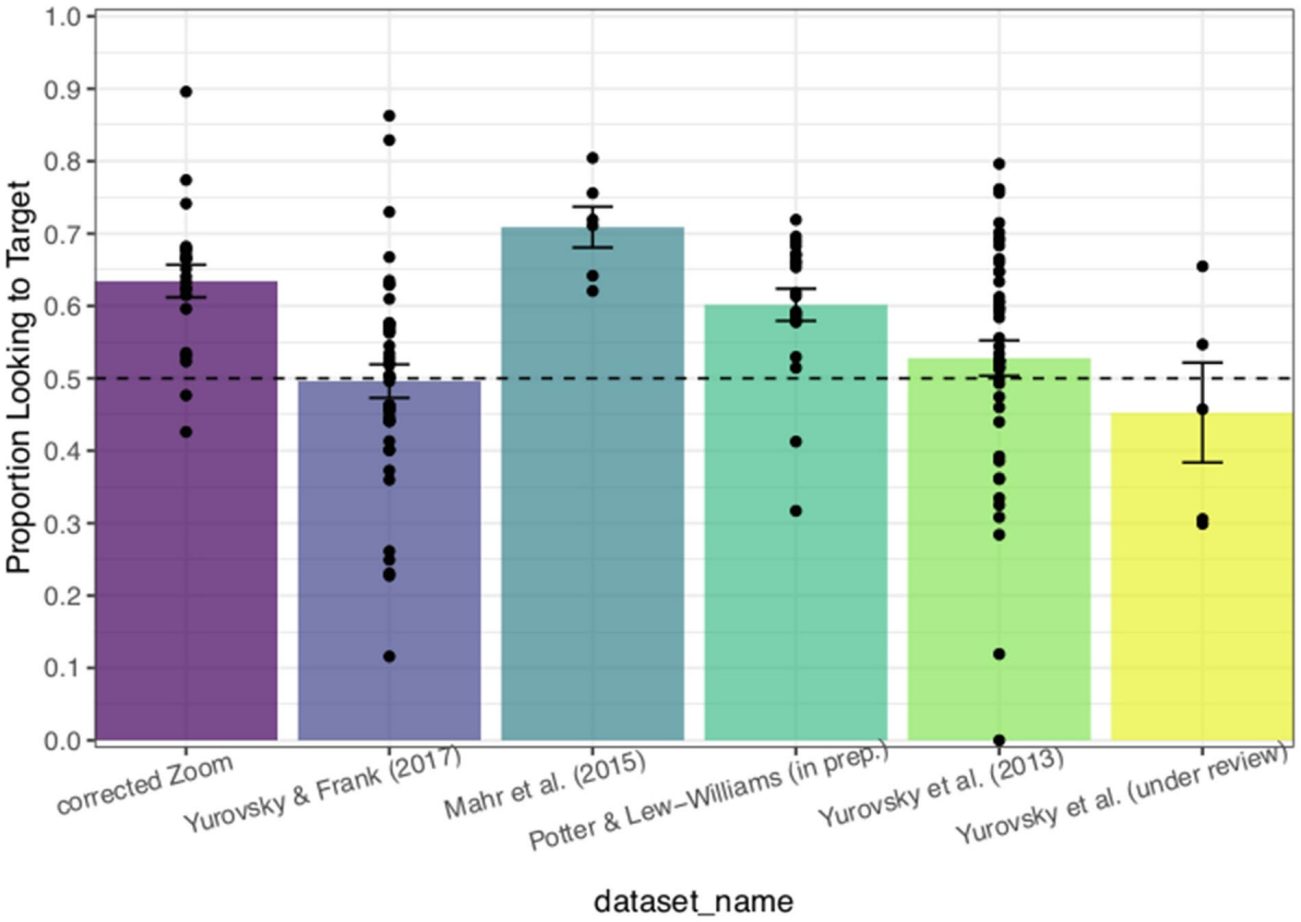
Proportion of looks to the target (accuracy) by study, comparing Study 1 to selected Peekbank studies. Chance looking behavior is denoted by the dashed line at 0.5 on the *y*-axis. Error bars represent SEM.

On average, infants’ accuracy on the online LWL task was significantly greater than chance [*b*=0.162; *t*(1, 76.570)=4.664; *p*<0.05]. Accuracy on the online LWL task was significantly different from accuracy in the in-lab data collected in Yurovsky and Frank (2017) [*b*=−0.151; *t*(1, 165.880)=−3.417; *p*<0.003], Yurovsky et al. (2013) [*b*=−0.160; *t*(1, 237.895)=−3.990; *p*<0.05], and Yurovsky et al. (under review) [*b*=−0.222; *t*(1, 296.866)=−2.900; *p*=0.012]. As can be seen in Figure 4 accuracy on the online task is significantly greater (*M*=0.634) than accuracy on the three in-lab tasks (*M*=0.496, *M*=0.528, and *M*=0.453, respectively). The range of in-lab familiar word recognition accuracy in Figure 4 suggests that the data collected *via* Zoom is feasible and valid.

### Study 1 Discussion

In Study 1, we evaluated the timing precision of online experimenter-moderated eye-gaze measures of LWL. Approximately 88% of the trials in the were usable, which constitutes similar rates of data loss to in-person data collection (Wass et al., 2013; Venker et al., 2020; but also see Oakes, 2010 for a call for greater transparency in reporting eye tracking data). Data quality was not significantly impacted across a range of different internet speeds, suggesting that various levels of internet connectivity can be supported in online data collection using these paradigms. Although internet connectivity did not preclude participation, it did contribute to immense frame rate variability across participant video recordings. Individual variability in timing can be corrected during data preprocessing using group-level average frame rates. Using this correction technique, we can account for differences in home testing conditions that are not typically seen in the lab that utilizes a single set of technical equipment. Taken together, these results suggest that looking-time behaviors can be captured *via* videoconferencing across a variety of home-set ups.

Across participants, there were differences in RT; however, the corrected and uncorrected RTs from the present sample were more similar to one another than they were to the in-lab RTs in the Peekbank data. This may be due to the differences in set-up for the online study administration and in-lab study administration. In Study 1, participants were seated in their caregivers’ lap like they would be in the lab, but were watching the study visuals occur on a much smaller screen and at a much closer distance than they would in-lab. The current design also tested each target word six times and the same images were seen multiple times, which may account for faster processing speeds.

In sum, the findings from Study 1 suggest that online data collection is feasible and yields high quality data, particularly when the data are adjusted to reflect frame rates. Experimenter-moderated online studies may be a way to collect more equitable and representative samples given that access to high-speed internet is not a requirement for participation. Families, who typically would not be able to attend in lab sessions due to scheduling, travel, or other barriers could join a 20min Zoom session to participate during a time and location that is convenient for them. Despite this theoretical benefit to online testing, Study 1 included a homogenous, predominantly White sample. Importantly, simply moving to a virtual platform did not ameliorate the issue of diversity in the participant sample. In Study 2, we test a recruitment method to increase the diversity of our participant pool for future online studies.

## Study 2: Online Recruitment

Study 1 provided preliminary promise that data collection using infant looking time measures was possible across several different home set-up variables. These results suggest that internet connectivity should not preclude participation in our experiment. Yet, the sample in Study 1 was homogenous and WEIRD despite being conducted online. Thus, simply administering the task in the home was not sufficient to recruit a more diverse, representative sample. This was likely due to the use of our existing participant database, developed for the local community, for Study 1. In Study 2, we examined whether targeting our recruitment efforts to locations with more diverse populations could provide a more representative sample for future online studies. At present, it is unknown whether ads targeting more diverse locations actually lead to more diverse potential research participants in developmental studies. The goal of this study was to determine whether microtargeting based on location can alter the demographics of respondents to our research advertisements.

### Methods

#### Participants

This study focused on caregivers who responded to Facebook advertisements that were microtargeted to display in two different cities. Upon clicking a sign-up link in the Facebook ad, caregivers voluntarily provided contact information that can be used to alert the family of future study opportunities. For analytic purposes, we considered a respondent to be a unique sign-up that included a child’s name and caregiver contact information. Fifty-one respondents were included in the data analyses (Site 1 *N*=14) for children that ranged in age from *in utero* and expected to be born in 2021 to 99-month. Eight additional respondents who signed up were excluded because they did not provide contact information. Note that we only collected information about names, ages, mailing addresses, and other contact information from respondents; we did not have IRB approval to collect any demographic information (e.g., race, SES) on our sign-up link. Thus, as noted below, we used census-tract data as a proxy to estimate demographic information about the caregivers who responded to our ad.

#### Materials

The ad featured two photos of participants and experimenters during study administration (Figure 1) (1) featuring two experimenters (one White, one Black) and a caregiver/child duo (both Black) and (2) featuring one experimenter (multiracial) and a caregiver/child duo (both White). These photos were selected based on work indicating that diversity in advertisements begets diversity in recruitment (Avery et al., 2004; Walker et al., 2012; Pechmann et al., 2020).

#### Procedure

This study focused on caregivers who responded to microtargeted Facebook advertisements that were directed at zip codes in two Midwestern cities in the same state with different demographic profiles. Site 1 has a predominantly Black population and Site 2, the catchment area for our in-lab studies, has a predominantly White population. We targeted the ad to a subset of zip codes within each city to ensure that the recruitment catchment areas were comparable in population size but varied on other key demographic features related to diversity (i.e., household income; see Table 2).

**Table 2:** Demographic and socioeconomic factors for the two target recruitment sites (set of zip codes targeted in the Facebook ad) as reported on the American Community Survey (ACS) 5-year estimates.

	Site 1	Site 2
Population	31,565.83	34,705.83
***Race and ethnicity***		
Hispanic	5.00%	10.00%
American Indian or Alaskan Native	1.00%	1.00%
Asian	6.00%	10.00%
Black or African American	68.00%	10.00%
Native Hawaiian or Pacific Islander	0.00%	0.00%
Other	3.00%	4.00%
White	26.00%	79.00%
***Socioeconomic factors***		
Cost of living index	97.18	98.35
Median *per capita* income	$39,479.83	$61,326.83
Percentage of children below the poverty line	39.00%	16.00%

*These locations vary on racial, ethnic, and socioeconomic (SES), but are comparable in population size*.

The ad targeted users aged 18–65+ with interests matching some or all of the following: family, motherhood, fatherhood, parenting, breastfeeding, childbirth, day care or early childhood education, job titles that included “science,” parents: new parents (0–12months), or parents with toddlers (01–02years).

#### Collecting Demographic Variables

Study 2 primarily focused on recruiting participants for future participation in online studies. The current respondents did not partake in any research. Thus, neither did they provide consent, nor did they contribute any data or demographic information. Upon sign-up, caregivers voluntarily provided contact information to be used to alert the family of their child’s eligibility to participate in a study. Facebook does not currently provide ad users with demographic features (other than age) about those who interact with their advertisement engagements. Thus, to assess the success of our ad in eliciting responses from more diverse populations, we relied on demographic metrics drawn from the American Community Survey (ACS) 5-year estimates (United States Census Bureau, 2020). We identified the US census tract for the home address of each sign-up we received. For each respondent, we use the demographic features (i.e., race, ethnicity, income, etc.) available for their *census tract* as a proxy for the likely demographic features of the participant. A US census tract accounts for one square mile of a geographic location. Thus, for Site 1, each percentage estimate reflects the proportion of people out of 6,188 people within a given area that identify with the demographic feature of interest, while Site 2 reflects the proportion of people out of 3,037 people within a given area (United States Census Bureau, 2020). For example, if 95% of people within a census tract within Site 1 identify as Black or African American this can be interpreted as approximately 5,879 out of 6,188 people within the census tract identify as Black or African American. We acknowledge that these data may not accurately represent the demographic characteristics of our individual respondents. However, the ACS demographic estimates allow us to empirically evaluate whether microtargeted Facebook ads resulted in sign-ups from groups of participants located in areas that are more diverse than those typically targeted for research participation.

### Results

#### Facebook Ad Results

Facebook estimated that our microtargeted advertisement ($400.00 USD total for a 14-day run) reached 13,392 people and that there were 577 interactions with the advertisement (see Table 3). Facebook defines interactions to include shares, likes, comments, and clicks. Of these interactions, 159 of them were clicks on links included in the ad (lab website link and sign-up link), which resulted in 59 new participant sign-ups. Metrics revealed that the ad was primarily presented in FB mobile app feeds (12,956 people out of 13,392 total reach).

**Table 3:** Facebook ad reach metrics.

Facebook metric	Estimated value
Total reach	13,392
Percent women	88.9%
Engagements	577
Reactions	215
Link clicks	159
Shares	14
Comments	5
Sign-ups	59

#### Demographics by Targeted Site Location

The aim of the microtargeted Facebook ad was to provide a more diverse pool of participants than typically generated by local convenience sampling. Because we did not have direct information about the demographics of our sample (as noted earlier, these data represent sign-ups for future studies rather than consented participants), we estimated the demographics of the respondents using census tract-level data. We identified the census tract number for each unique address provided at sign-up which resulted in 35 unique tracts. Two additional tracts were excluded for being located out of the target state. Each census tract was then coded as located in either Site 1 (13 tracts) or Site 2 (22 tracts). To evaluate the diversity of each group of potential participants (Site 1 vs. Site 2), we queried the ACS 5-year estimates for four factors related to diversity: racial makeup, ethnic background, educational attainment for the population over 25-years-old, and median household income for each of the census tracts. To determine whether there were potential differences in the demographics of respondents from each site, we ran linear regression models using the lmSupport package (Curtin, 2018) in R (Version 1.2.1335; R Core Team, 2019). We report the results of the regression analyses for each diversity metric, separately.

##### Racial Makeup

We evaluated the potential racial diversity of our respondents by comparing the racial makeup according to the ACS estimates derived from respondents’ census tracts between the two site locations. We predicted that a higher proportion of respondents from census tracts located in Site 1 would belong to more diverse racial categories than those from Site 2, where we expected that the majority of the respondents would identify as White. To test this hypothesis, we computed proportion of the population (number of people within a respondent’s census tract that identify as a racial category/total population within the census tract) that identified as American Indian or Alaskan Native, Asian, Black or African American, Native Hawaiian or Pacific Islander, White, or Other. Thus, for each respondent we calculated six proportions, corresponding to each of the racial categories from their census tract data. We fit a linear model regressing these proportions on race (dummy coded with White as the reference group), site (centered, coded Site 1=−0.5 and Site 2=0.5), and their interaction. The results of the linear model are reported in Table 4. Given that the variable race was dummy coded, each estimate indicates whether there is a significant difference in the average proportion of White people compared to each of the other racial categories (i.e., proportion of White people compared to the proportion of Black people). All values of *p* were corrected for multiple comparisons using the Holm-Bonferroni approach (Holm, 1979).

**Table 4:** Results of regression analyses by demographic metric.

Demographic metric	*b*	*F*	*p*	*df*	*R* ^2^
*Race*				288	0.845
Site	0.469	189.64	0.000		
Asian vs. White	−0.204	24.76	0.000		
Black or African American vs. White	0.375	84.03	0.000		
American Indian or Alaskan Native vs. White	−0.246	36.09	0.000		
Native Hawaiian or Pacific Islander vs. White	−0.250	37.27	0.000		
Other vs. White	−0.231	31.78	0.000		
Asian vs. White * Site	−0.434	81.06	0.000		
Black or African American vs. White * Site	−0.967	402.11	0.000		
American Indian or Alaskan Native vs. White * Site	−0.469	94.76	0.000		
Native Hawaiian or Pacific Islander vs. White * Site	−0.472	95.65	0.000		
Other vs. White * Site	−0.471	95.46	0.000		
*Ethnicity*				78	0.969
Site	0.000	0.000	1.000		
Ethnicity	−8.965	903.474	0.000		
Site * Ethnicity	0.061	2.723	0.103		
*Education*				240	0.476
Site	0.135	19.025	0.000		
Associate’s degree vs. Graduate degree	0.001	0	1.000		
Bachelor’s degree vs. Graduate degree	0.050	1.833	0.531		
High school diploma or less vs. Graduate degree	0.358	92.991	0.000		
Some college vs. Graduate degree	0.188	25.522	0.000		
Associate’s degree vs. Graduate degree * Site	−0.123	7.854	0.022		
Bachelor’s degree vs. Graduate degree * Site	0.026	0.344	1.000		
High school diploma or less vs. Graduate degree * Site	−0.347	62.699	0.000		
Some college vs. Graduate degree * Site	−0.232	27.952	0.000		
*Income*					
Site	23,428	14.13	0.000	48	0.227

**Figure 5:**
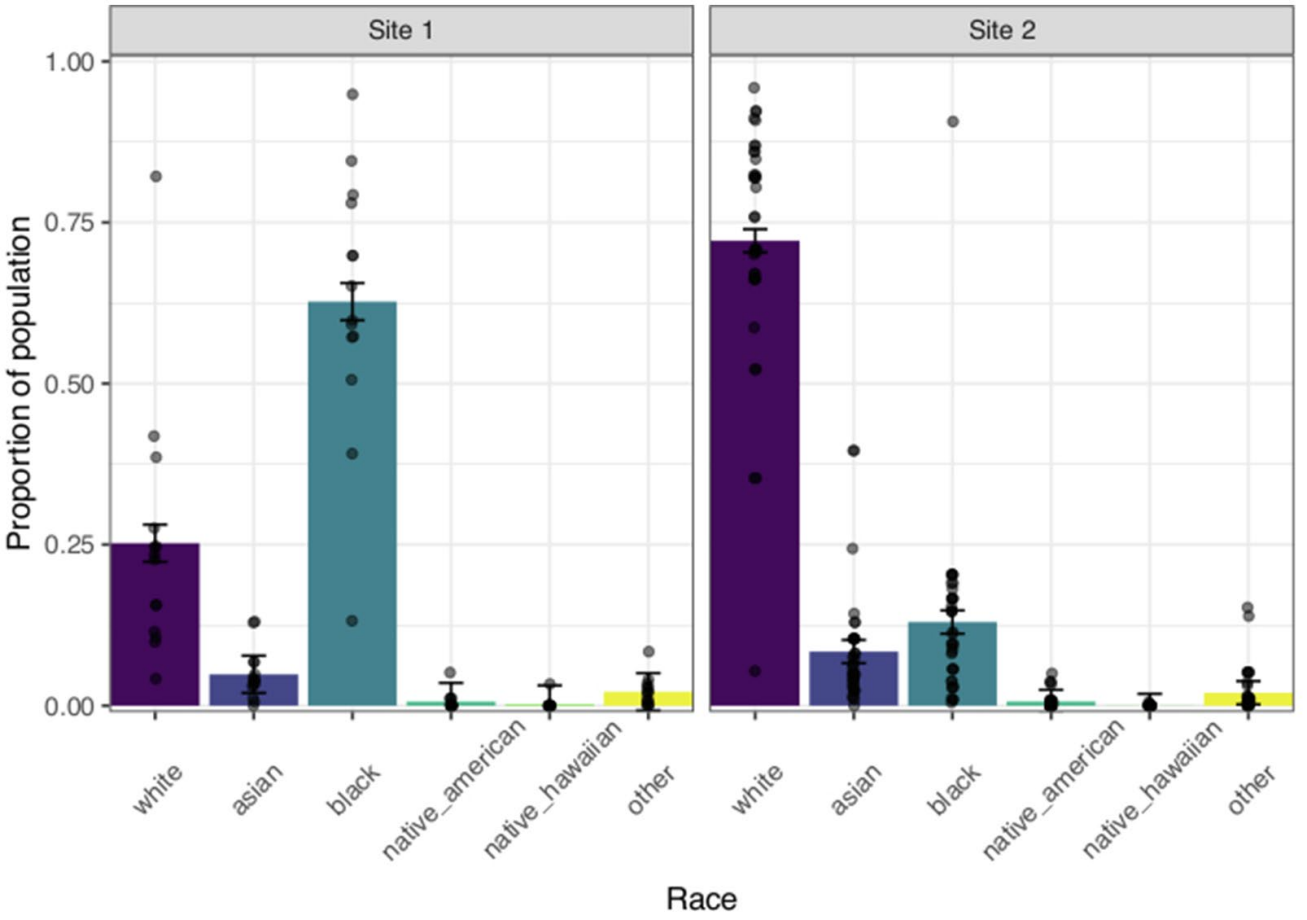
Racial makeup by site location for respondents. Each bar represents the average proportion of each site’s population that identifies as a particular racial category. The first panel shows mean values for Site 1 and the second panel shows mean values for Site 2. Each data point represents the proportion of the population that identifies as a particular racial category for individual respondents’ census tracts. Error bars indicate SEM.

Overall, a significantly higher proportion of residents across both sites identified as White (*M*=0.590) as compared to Black or African American (*M*=0.27), Asian (*M*=0.070), or American Indian or Alaskan Native (*M*=0.010). Importantly, there was also a significant race by site interaction [*F*(5, 288)=80.704, *p*<0.05, *n*^2^*p*=0.584]. This significant interaction indicates that the mean proportion of White people compared to the proportion of people that identified as each of the other racial categories differed depending on Site location (Figure 5). Specifically, compared to Site 2, respondents whose census tracts were located in Site 1 had a significantly higher proportion of people that identified as Black [*b*=−0.967, *F*(1, 288)=402.11, *p*<0.05], Asian [*b*=−0.434, *F*(1, 288)=81.06, *p*<0.05], American Indian or Alaskan Native [*b*=−0.469, *F*(1, 288)=94.76, *p*<0.05], Native Hawaiian or Pacific Islander [*b*=−0.472, *F*(1, 288)=95.65, *p*<0.05], or Other [*b*=−0.471, *F*(1, 288)=95.46, *p*<0.05; Table 4]. The Facebook ad specifically targeted a predominantly Black location (Site 1) and a predominantly White location (Site 2). The results from the regression analysis suggest that the respondents from Site 1 represent a more diverse set of potential participants. As can be seen in Figure 5, respondents’ census tracts in Site 1 included a higher proportion of Black or African American people (*M*=0.627) compared to White people (*M*=0.252), while respondents’ census tracts in Site 2 included a smaller proportion of Black or African American people (*M*=0.130) compared to White people (*M*=0.722).

##### Ethnic Background

We examined ethnic diversity between the two targeted sites by conducting a parallel analysis to the analysis of racial diversity. We calculated the proportion of the population that identified as Hispanic or non-Hispanic for each of the census tracts (number of people within a respondent’s census tract that identify as Hispanic or non-Hispanic/total population within the census tract). Given the target site demographics (Table 2), we predicted that Site 2 would have a slightly higher proportion of Hispanic respondents than Site 1. We tested this hypothesis by fitting a linear model predicting the proportion of the population from ethnicity (centered, coded Not Hispanic=−0.5, Hispanic=0.5), site (centered, coded Site 1=−0.5, Site 2=0.5), and their interaction. The results of the regression indicate a significant effect of ethnicity [*b*=−8.965, *F*(1, 78)=903.474, *p*<0.05]. On average, there was a greater proportion of non-Hispanic respondents (*M*=0.930) than Hispanic respondents (*M*=0.07) across both sites. No other effects were significant (Table 4).

##### Educational Attainment

To assess differences in educational attainment between the two sites, we used the ACS 5-year estimates to separately compute the proportion of the population over 25 that has received varying levels of education (High School Diploma equivalent or less, some college, Associate’s degree, Bachelor’s degree, or Graduate degree). The ACS estimates report educational attainment status for different age bands including 18–24 and 25+. We selected the estimates for the population over age 25 because the Facebook ad primarily reached an audience age between 25 and 44. For each respondent’s census tract, we calculated five proportions (number of people within a respondent’s census tract that attained an education level/total population within the census tract), corresponding to each of the education levels. We regressed the calculated proportions on educational attainment (dummy coded with Graduate degree as the reference group), site (centered, coded Site 1=−0.5, Site 2=0.5), and their interaction. Site 2 represents a typical University-based convenience sample and therefore we expected that respondents from Site 2 would have a higher proportion of highly educated people compared to Site 1.

**Figure 6:**
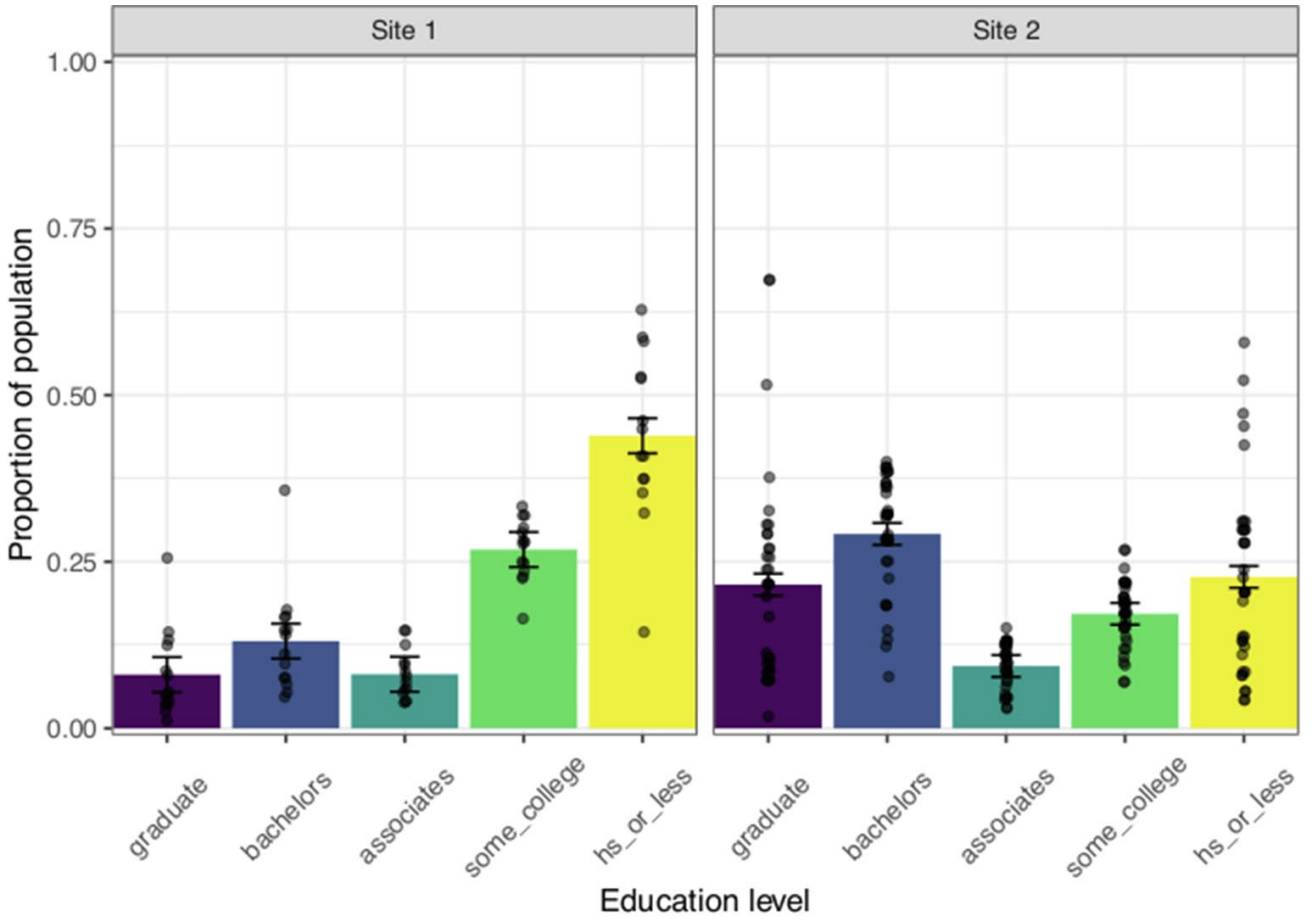
Educational attainment by site location for respondents. Each bar represents the mean proportion of the population that has attained each education level for Site 1 (panel 1) and Site 2 (panel 2). Each data point represents the proportion of the population that has reached each education level for individual respondents’ census tracts. Error bars represent the SEM.

The full results of the model analyzing educational attainment can be seen in Table 4. The variable educational attainment was contrast coded with Graduate degrees as the reference group, which resulted in four different comparisons. Each estimate indicates whether the proportion of the population that received a Graduate degree differs significantly from the proportion of the population that received each of the other levels of education (i.e., proportion of people that received a Graduate degree compared to the proportion of people that received a high school degree). Collapsing across sites, a higher proportion of individuals living in the respondents’ census tracts reported having a High School diploma or less (*M*=0.290) or attended some college (*M*=0.200) compared to a Graduate degree (*M*=0.180). There was also a significant site by educational attainment interaction [*F*(4, 240)=25.644, *p*<0.05, *n*^2^*p*=0.299]. As shown in Figure 6, Site 1 had a greater proportion of people that received a High School Diploma or less (*M*=0.439) than a Graduate degree (*M*=0.081) [*F*(1, 240)=62.299, *p*<0.05] compared to Site 2 (*M*=0.227 and *M*=0.216 for High School Diploma or less and Graduate degree, respectively). The same pattern of results can be seen in Figure 6 for the proportion of people located in Site 1 that attended some college (*M*=0.268) rather than those who received a graduate degree (*M*=0.081) as compared to Site 2 (*M*=0.172 and *M*=0.216 for some college and Graduate degree, respectively). The analysis of educational attainment supports our prediction that targeting a more diverse location can provide a sample of participants that have a wider range of educational backgrounds than typically seen in University-based samples.

##### Household Income

We identified the median household income estimates from the ACS for each census tract to provide a metric of socioeconomic diversity for respondents from the two sites. We predicted that Site 2 would have a higher median income than Site 1, reflecting the typical wealthy convenience sample. To investigate this hypothesis, we fit a linear model predicting median household income from site location. Site location significantly predicted median income [*b*=23,428, *F*(1, 48)=14.13, *p*<0.05, *n*^2^*p*=0.227]. Site 2 had a higher median income (*M*=68,997.33) than Site 1 (*M*=45, 569.07). These results indicate that targeting Site 1 resulted in a sample of respondents who are likely to be more economically diverse than would have been possible had we only targeted the local convenience sample.

### Study 2 Discussion

Study 2 investigated whether targeting a Facebook advertisement to a more diverse location would provide a more representative pool of participants for future online research. We directed microtargeting Facebook advertisements toward two locations: a diverse urban community and a location proximal to our university that reflects typical local convenience sampling. The advertisement had high engagement and provided 59 new sign-ups across the two site locations. Importantly, the analyses of our census tract-based diversity metrics suggest that the 14 respondents from Site 1 were likely to be more diverse in racial, educational, and economic backgrounds than the 37 respondents from Site 2. Our results lend credence to the potential benefits of recruiting representative samples for online studies using targeted Facebook ads. Further, these results suggest that widening the net of recruitment to more diverse locations can create a pool of participants for online studies who are more demographically representative than is possible for in-lab studies that are limited by the diversity of the local population. It remains to be seen, however, whether greater diversity in families who respond to our ads will lead to greater diversity in the families who eventually choose to participate in our studies.

## General Discussion

The present work demonstrates (1) the viability of the Zoom platform for experimenter-moderated looking time studies using LWL paradigms with infants, (2) the feasibility of online participation regardless of internet speed, and (3) the effectiveness of microtargeted Facebook ads for recruiting a more diverse group of potential participants. Overall, the current research demonstrates not only just the feasibility of running studies with infants online with this paradigm, but also addresses some of the immediate concerns surrounding recruitment diversity and data quality.

Caregivers were able to appropriately set-up their computer for the study with the virtual aid of the experimenter and deploy the experiment themselves while the experimenter recorded and stored the participant data. This method did not sacrifice data quality and was easy to administer. Although access to high-speed internet was a paramount concern prior to online data collection, the current study suggests that internet connectivity does not significantly reduce data quality. Lower speed internet can impact the experiment presentation, but the Zoom recording captures these perturbations. For example, the experiment did not display the first trial when running the study using 2G internet. The experimenter-facing Zoom recording reflected this presentation error and looking behavior was not coded during the missed trials. We also anticipated that internet speed would significantly impact the validity of the timing of some participants’ data. However, we were able to accommodate this variability by individually adjusting the frame rate for each trial prior to data analysis. Together, these findings demonstrate that online data collection can yield similar results to in-lab studies without significant restrictions due to participant internet connectivity.

Virtual study administration is accompanied by concerns regarding equity in internet access. For optimal study administration and for the clearest data quality, faster internet speeds are optimal; however, this does not mean that slower internet speeds preclude participation. Participants in the sample had varied internet speeds, but that did not prohibit them from participation. As demonstrated by our internet speed testing results, there is minimal data loss at even the slowest internet speed, and the data loss that is incurred is present on both the participant and experimenter sides.

One disadvantage to online research using Zoom is that people cannot participate on tablets and smartphones, whereas TheChildLab can be used on these devices (Sheskin and Keil, 2018). Experimenter-moderated Zoom-based eye-gaze tasks like Study 1 require a desktop computer or laptop with a web camera and internet access to participate. The screen sharing function on Zoom does not allow for simultaneous screensharing and video sharing on tablets or smartphones. These constraints will prevent a segment of the population from having access to participating in research like Study 1. According to the National Center for Education Statistics (2020), 6% of 3- to 18-year-old only have home access to the internet *via* smartphone, with an additional 6% of children having no internet access at home. Most of the children without access to the internet *via* a device other than a smartphone are from minority groups, have parents with the equivalent of a high school diploma or less, and are from the lowest quarter of all family incomes (National Center for Education Statistics, 2020). In other countries, lack of access may be substantially greater. Lourenco and Tasimi (2020) suggest several ways to combat these limits on research participation, including mobile laboratory set-ups to go into communities with less internet access and providing mobile hotspots to participant families for participation. These approaches may facilitate recruiting representative participant samples, as 12% of the child participant population is currently unreachable *via* the Zoom videoconferencing online methodology.

Online recruitment is not enough to check the diversity box, as is evident in the highly non-representative sample in Study 1. Online recruitment efforts require intentionality in making decisions on the locations to target and the materials included in the ads. Microtargeted Facebook (and Instagram) ads work for caregiver, and subsequent infant, recruitment. The results of Study 2 suggest that we may have reached more diverse respondents *via* recruitment efforts focused on specific area codes. However, because we have not yet enrolled these respondents in studies, additional research is needed to verify that these recruitment efforts subsequently result in more representative study participants. Further, the microtargeted Facebook ads used in the current study depicted a White infant with a multiracial researcher and a Black infant with both a Black and a White researcher. These advertisement design decisions may have increased the level of response by non-White caregivers, perhaps because they saw people that look like themselves and their child(ren) represented in a research setting. Indeed, findings from the marketing literature demonstrate positive relationships between the amount of diversity presented in recruitment materials and recruitment of more diverse job candidates (Avery et al., 2004; Walker et al., 2012).

### Limitations and Future Directions

Study 1 demonstrates that experimenter-moderated LWL tasks are feasible *via* the Zoom platform. However, the conclusions that we can draw about the timing of fixations is limited by the comparisons we can draw. Because we do not have an identical in-lab task to which we can compare the timing data, we compared our data to other in-lab LWL studies reported on Peekbank (Zettersten et al., 2021). While this is a helpful comparison, many features diverge between our task and these extant data (e.g., number of trials testing each word). Additionally, the set-ups of virtual and in-lab studies differ tremendously in the positioning of the child relative to the screen, as well as the size of the screen on which the study is administered. If administered in our current lab set-up, this study would have been presented on a 55-in Toshiba LCD television with participants seated on their caregiver’s lap 3 feet away from the screen. In the virtual experimenter-moderated version reported in Study 1, the task was administered on a 13- to 15-in computer screen with the participants approximately 1 foot away from the screen. In both environments, objects on the screen are evenly spaced on the left and right of the screen, but the size of the objects and the distance between them differs as a function of the size of the monitor. This may account for some of the looking time differences between Study 1 and the Peekbank comparison – the distance between objects impacting the amount of time it takes to complete a saccade.

In Study 2, recruitment efforts *via* selected diverse photos and microtargeting diverse zip codes led to respondents from more diverse locations, but this does not necessarily beget diverse study participants. In line with what we had predicted, there was more diversity in the respondents from Site 1, though the overall number of respondents from Site 1 was less than half than the number of respondents from Site 2. This aligned with our concern of whether people from a more diverse area, and an area that is non-local to the University, would be willing to sign up to participate in an online study due to historical mistrust of research. In the future, additional ad specificity would allow a better understanding of the degree to which microtargeted recruitment increases the diversity of participant samples. This would also supply added insight into the remaining barriers for diverse participation. Microtargeted ads using the Facebook ad platform are accessible from mobile devices and tablets, though a mobile-device is not compatible with the present experimenter-moderated study administration. The requirement of a computer with a web camera and internet access places an added burden on the participants’ families and may be a hindrance to study participation, despite sign-up interest.

In sum, conducting studies online provides a wider range of participant families the opportunity to partake in research, without researchers sacrificing data quality due to internet connectivity. The Zoom videoconferencing platform is widely available to caregivers and provides an easy avenue for experimenter-moderated eye-gaze studies using LWL. Moving forward with online data collection requires intentionality on the part of the researchers to ensure they are recruiting diverse participants by using thoughtfully constructed recruitment materials, including the photos and language used. These efforts, combined, allow data collection to continue at a distance, and move us closer to samples that are more representative of the demographics of the population. The present work demonstrates not only the success, but also the feasibility of these efforts.

## Data Availability Statement

The datasets presented in this study can be found in online repositories. The names of the repository/repositories and accession number(s) can be found at: https://osf.io/e9a8y/?view_only=f74b7fbdcfc04cdda13b479cf08a4c67

## Ethics Statement

The studies involving human participants were reviewed and approved by The University of Wisconsin-Madison Educational and Social Behavioral Science Institutional Review Board. Written informed consent to participate in this study was provided by the participants’ legal guardian/next of kin, and obtained from the individual(s) and minor(s)’ legal guardian/next of kin, for the publication of any potentially identifiable images or data included in this article.

## Author Contributions

DB and HW designed the study with critical insight from JS and wrote the manuscript. HW performed the research and analyzed the data. DB, HW, and JS contributed to revisions of the manuscript. All authors contributed to the article and approved the submitted version.

## Funding

This material is based upon work supported by the National Science Foundation Graduate Research Fellowship Program under Grant No. DGE‐1747503 to DB. Any opinions, findings, and conclusions or recommendations expressed in this material are those of the author(s) and do not necessarily reflect the views of the National Science Foundation. This work was also supported by grants from NICHD awarded to JS (R37HD037466) and the Waisman Center (U54 HD090256).

## Conflict of Interest

The authors declare that the research was conducted in the absence of any commercial or financial relationships that could be construed as a potential conflict of interest.

## Publisher’s Note

All claims expressed in this article are solely those of the authors and do not necessarily represent those of their affiliated organizations, or those of the publisher, the editors and the reviewers. Any product that may be evaluated in this article, or claim that may be made by its manufacturer, is not guaranteed or endorsed by the publisher.

## Footnotes

^1^The Databrary Project is accessible to institutionally affiliated researchers at databrary.org

^2^The OSF repository containing the stimuli for the larger project can be found at https://osf.io/nuecw/?view_only=0208ac75881148c9b602e22520a1bdc1

^3^GIMP is an open-source editing software and is accessible at https://www.gimp.org/
